# The Role of microRNA in Pathogenesis, Diagnosis, Different Variants, Treatment and Prognosis of Mycosis Fungoides

**DOI:** 10.3389/fonc.2021.752817

**Published:** 2021-12-13

**Authors:** Pengfei Wen, Yao Xie, Lin Wang

**Affiliations:** Department of Dermatovenerology, West China Hospital, Sichuan University, Chengdu, China

**Keywords:** microRNA, Mycosis fungoides, pathogenesis, diagnosis, treatment, prognosis

## Abstract

Mycosis fungoides (MF) is the most common type of cutaneous T-cell lymphoma (CTCL), accounting for approximately 50% of all CTCLs. Although various molecular changes in MF have been described in existing studies, no obvious disease-specific changes have been found thus far. microRNAs (miRs) are short, noncoding RNA molecules that play roles in the post-transcriptional regulation of oncogenes and tumor suppressor genes in various diseases. Recently, there has been rapidly expanding experimental evidence for the role of miRs in the progression, early diagnosis, prognosis prediction for MF. Efforts to improve early diagnosis and develop personalized therapy options have become more important in recent years. Here, we provide an overview and update of recent advances regarding miRs associated with MF. Furthermore, we provide insights into future opportunities for miR-based therapies.

## Introduction

Mycosis fungoides (MF) is the most common type of cutaneous T-cell lymphoma (CTCL), accounting for approximately 50% of all CTCLs. The clinical manifestations usually present with erythematous patches and plaques with an indolent course and may slowly progress to tumors (1). Similar to inflammatory dermatosis, the lesions of MF patients in the stable stage can last for decades with a favorable prognosis. The period between the onset of skin lesions and diagnosis can vary from several months to years, with some patients’ diagnoses delayed by more than four decades (2). However, in a proportion of early MF patients, the disease progresses rapidly and enters a more advanced stage with visceral spread, requiring more aggressive treatment regimens (3). Unfortunately, it is currently difficult to distinguish inflammatory dermatosis from early MF and identify patients with favorable or poor prognosis before treatment. Therefore, new approaches are needed to improve the accuracy of the early diagnosis and predict the prognosis of MF.

**Figure 1:**
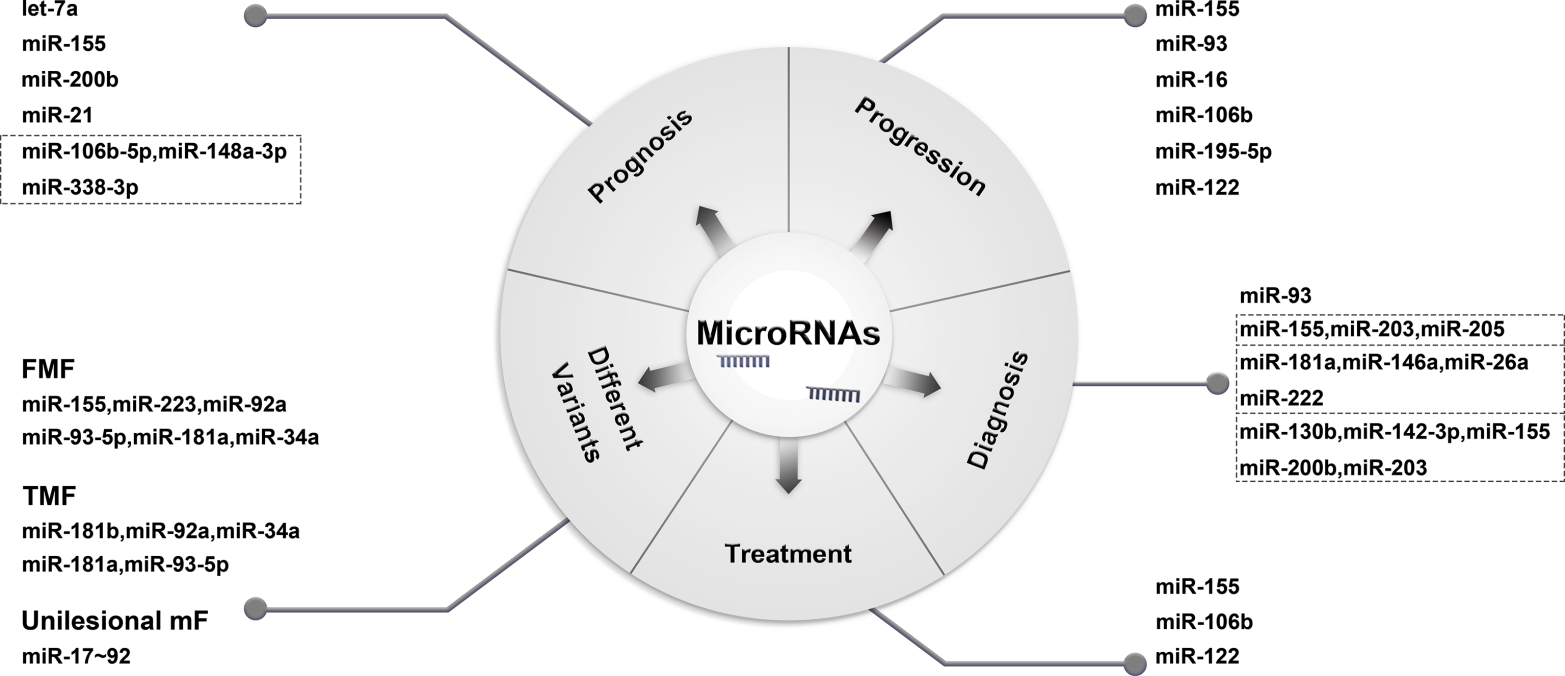
Functions of miR in MF.

Although various molecular changes in MF have been described in existing studies, including chromosomal, genomic, and gene expression aberrations; no obvious disease-specific changes have been found thus far (4). MF is a clonal disorder with specific T-cell receptor (TCR) gene rearrangement. In addition to clinical and histological findings, TCR clonality testing is a helpful adjunct diagnostic method (5). However, the sensitivity of TCR rearrangement detection varies greatly according to different clinical stages, methods of assay and primer design (6–8). Therefore, there is still an urgent need to explore more specific and sensitive biomarkers to help us better understand and manage MF. MicroRNAs (miRs) are short noncoding RNA molecules that play roles in post-transcriptional regulation by binding to RNA-induced silencing complexes and controlling physiological and pathological processes in various diseases (9). In addition, miRs play important roles in tumorigenesis and function as oncomiRs or tumor suppressors by regulating the levels of oncogenes or antioncogenes (10–12). The miR expression profiles in MF have been studied extensively and have shown a high correlation with disease progression, prognosis, and response to treatment (**Table 1**) (11–42). In this review, the function and molecular mechanism of miRs in the progression, diagnosis, variants, prognosis, and treatment of MF are discussed in detail (**Figure 1**).

**Table 1:** miR expression profiles of available studies in MF.

MiR ID	Expression	Functional role	Reference
miR-155	upregulate	diagnosis, progression, different variant (FMF), prognosis prediction, treatment	(11, 13–28)
miR-203	downregulate	diagnosis	(16, 19, 24)
miR-205	downregulate	diagnosis(racial differences)	(19, 24)
miR-92a	upregulate	progression, differential diagnosis, different variant(FMF, TMF)	(15, 23)
miR-93-5p	upregulate	different variant(FMF,TMF)	(13, 29)
miR-93	upregulate	progression, differential Diagnosis, different variant(FMF),treatment	(12, 15, 17, 24, 29–31)
	downregulate	early diagnosis	(32)
miR-19b	upregulate	different variant(FMF)	(23)
miR-34a	upregulate	different variant(FMF)	(13)
miR-223	upregulate	different variant(FMF), treatment response prediction	(13, 33)
miR-191	upregulate	treatment response prediction	(33)
miR-342	upregulate	treatment response prediction	(33)
miR-181	upregulate	progression	(30)
miR-181a	upregulate	diagnosis, progression, different variant(FMF,TMF)	(13, 30, 34, 35)
miR-181b	upregulate	different variant(TMF)	(13)
miR-338-3p	upregulate	prognosis prediction	(36)
miR-148a-3p	upregulate	prognosis prediction	(36)
miR-106b	upregulate	progression, prognosis prediction	(14, 36, 37)
miR-106b-5p	upregulate	progression, prognosis prediction	(36)
let-7a	downregulate	prognosis prediction	(15)
miR-17~92	upregulate	progression, different variant(unilesional MF)	(38)
miR-243	upregulate	diagnosis	(16)
miR-22	downregulate	progression	(39)
miR-200b	upregulate	diagnosis, good prognosis	(16)
miR-146a	upregulate	diagnosis	(34)
miR-222	not mentioned	diagnosis	(34)
miR-26a	not mentioned	diagnosis	(34)
miR-142-3p	upregulate	diagnosis	(16)
miR-130b	upregulate	differential diagnosis	(16)
miR-195-5p	downregulate	progression	(37, 40)
miR-122	upregulate	progression, treatment	(41)
miR-15a	downregulate	progression, prognosis prediction	(17)
miR-16	upregulate	progression, clinical course prediction	(15, 32)
	downregulate	diagnosis, progression, clinical course prediction	(17)
miR-21	upregulate	differentia diagnosis, clinical course prediction	(14)

FMF, Folliculotropic mycosis fungoides; TMF, mycosis fungoides with larger cell transformation.

## The Function of miR and Clinical Application in Tumor

miR is a short, noncoding RNA molecule that can regulate mRNA expression at the post-transcriptional level and is widely found in viruses, plants and animals. miR is broadly involved in a variety of physiological and pathological processes, and dysregulated miR expression is related to cancer initiation and progression (10). It does not encode functional proteins but can degrade or inhibit protein translation by means of complementary pairing with target mRNA and eventually inhibit specific gene expression (43).

In recent years, many studies have shown that miR is involved in the occurrence and development of tumors and has a broad clinical application space in the diagnosis and treatment of human cancers and hematological malignancies. Specifically, miR in serum or tumor tissue samples can be used as potential tumor markers for early diagnosis, such as breast cancer and B-cell lymphoma (44–46). In addition, studies have shown that miR can be used as a specific molecular target for targeted therapy (9, 47). Currently, miR mimics and miRNA inhibitors in the preclinical phase of drug development have shown potential as novel therapeutic drugs in tumor-treating fields.

## miR and Cutaneous T-Cell Lymphoma (CTCL)

CTCL comprises a heterogeneous group of disorders with variable clinical presentations, histological features, and prognoses. Growing evidence demonstrates that miR is involved in the development and progression of CTCL (11, 16, 39, 48–50). There are significant similarities and differences in the expression of miR among different variants of CTCL. For instance, miR-155, which was first identified as abnormally expressed in CTCLs, can be used as an oncogenic driver to promote tumor growth in both MF and anaplastic lymphoma kinase (ALK)-negative anaplastic large-cell lymphoma (ALCL) (18, 51). There exists a significant difference in the miR expression profile between tumoral MF, erythrodermic MF and the more aggressive leukemic variant of CTCL—Sezary syndrome (SS)—such as the miR-155, miR-21, miR-93, miR-195–5p, and miR-17/92 (14, 15, 52, 53). Additional, Dercer expression have been served as an molecular marker in MF and might be of clinical relevance in MF, lymphomatoid papulosis and primary cutaneous CD4-positive small/medium T-cell lymphoma (54). To date, the mechanism of miR dysregulation in CTCL has not been fully elucidated and recognizing the abnormal expression of miR among different subgroups of CTCL variants, especially MF, is particularly important for elucidating the pathogenesis, early diagnosis, and identification of new therapeutic targets of CTCL.

## miR and MF

### miR Could Be a Gene Regulator in the Pathogenesis and Progression of MF

The molecular pathogenesis of MF remains limited. miR may function as an oncogenic or tumor suppressor and contribute to the pathogenesis and progression of MF through interactions with specific target genes.

miR-155 acts as an oncogenic miR and is overexpressed in multiple solid tumors and B-cell lymphoma (55–57). Currently, it is one of the most intensively studied miRs in MF. Significant upregulation of miR-155 and miR-92a in tumoral MF was first observed by Van Kester et al. in 2011 (15). Subsequent studies further identified the overexpression of miR-155 in both early and advanced MF, and the expression level of miR-155 in biopsy samples increased with increasing clinical stage (17, 18, 26). *In vivo* and *in vitro* experiments confirmed that miR-155 plays an important role in the development of MF and contributes to tumor growth by decreasing G2/M arrest and apoptosis (20). Additionally, previous studies indicated that the JAK/STAT5 pathway can promote the expression of miR-155 and promote the proliferation, growth, and survival of malignant MF cell lines *in vitro* (11). Interestingly, microbes have also been implicated in disease progression in CTCL (58, 59). A recent study showed that S. aureus and its enterotoxins might enhance miR-155 expression and promote disease progression by stimulating the expression of post-transcriptional regulators of malignant T cells (21).

Moreover, miR-93 has been described as an oncogene miR that can prevent apoptosis and promote tumor cell survival in various cancers (12, 31). miR-93 is overexpressed in advanced MF compared with inflammatory dermatosis and functions in the progression of MF (15, 17, 24, 30). Gluud et al. found that miR-93 can interfere with the expression of tumor suppressor cyclin-dependent kinase inhibitor 1 (p21) in MF tumor T cell lines. In turn, the expression of the p21 protein was significantly increased in cells (MF2059 and MF3675) transfected with a miR-93–5p inhibitor, resulting in a 20–30% decrease in the proliferation of malignant T cell lines (29). In addition, Katona et al. observed a trend toward a loss of PTEN expression with histological progression of MF (60). The tumor suppressor gene PTEN is a known target of the miR-93–5p and miR-181 families (61, 62). However, contrary to the findings of previous studies, a recent study on miR-93 found that the expression of miR-93 was significantly downregulated in both early and advanced MF compared with normal and eczema cases (32). Therefore, further studies with larger cohorts of MF patients are needed to explore the role of miR-93 in the progression of MF.

In addition to miR-155 and miR-93, miR-16 dysregulation may also play a certain role in MF progression. Maj et al. found that the decrease in miR-15a and miR-16 is related to the development of advanced MF (17). This finding is consistent with the results of most previous studies regarding miR-16 as a tumor suppressor in various tumors, including pituitary adenomas and chronic lymphocytic leukemia (CLL) (63, 64). However, van Kester et al. observed that miR-16 in tumoral MF was upregulated compared with inflammatory dermatosis (15). Similarly, additional evidence suggests that miR-16 was significantly upregulated in advanced MF compared with patients at early stage and could be used to predict aggressive clinical course (32). Thus, more studies are needed to explore the specific expression and biological function of miR-16 in MF.

Recent studies have indicated that miRs not only play an important role in MF tumors but also in the tumor microenvironment (TME). miR-106b expression was observed in dermal T lymphocytes in skin lesions from patients with MF, and the expression level increased as the disease progressed. miR-106b can promote tumor proliferation *in vitro* by inhibiting the tumor suppressor p21 and thioredoxin-interacting protein (37). In addition, the local expression of miR-106b in stromal cells indicated that miR-106b may play potential roles in the MF TME. Microenvironment-mediated changes in miR expression in tumor cells mediating progression have also been highlighted. Research has shown that cancer-associated fibroblasts (CAFs) can protect MF cells from doxorubicin-induced cell death and promote migration through the secretion of CXCL12 (65). However, the role of miRs in MF progression between tumor cells and matrix components in the TME has not been elucidated. The value of the TME in exploring the pathogenesis of MF deserves further investigation.

In addition to the abovementioned oncogenic miRs, the cumulative inhibition of multiple tumor suppressor miRs may lead to the downregulation of multiple signaling pathways driving the disease progression of MF (66). miR195–5p may play a role as a tumor suppressor in MF, and its inhibitory effect is related to disease progression (37). The upregulation of miR-195–5p inhibits cell cycle arrest through the downregulation of ADP-ribosylation factor-like protein 2 (ARL2), and low expression of miR-195–5p in MF skin lesions may promote disease progression (40). In addition, the role of miR-22 as a tumor suppressor in numerous solid tumors is widely accepted, and low miR-22 expression is associated with advanced stage and metastasis (67). *In vitro* studies have shown that miR-22 is significantly downregulated in malignant CTCL T cell lines (MyLa2059), and Jak3/STAT pathway-mediated inhibition of miR-22 may play a key role in CTCL pathogenesis and progression (39).

### miR Can Serve as Diagnostic Biomarkers in MF

Early clinical and pathological diagnosis of MF remains a challenge because of its clinicopathological similarity to benign inflammatory disorders, which may also exhibit clonal TCR rearrangement in some conditions (68, 69). Also, the lack of specific molecular markers that can reliably differentiate the malignant T-cells in MF from the reactive T cells in benign inflammatory disorders. Once entering the advanced stage, the median survival of patients with MF is only 1–5 years, with a 5-year survival rate of less than 15% (70). Therefore, the search for diagnostic molecules is still needed for early diagnosis and then timely treatment.

With the deepening of research, there is growing evidence to support the biomarker potential of miRs for the diagnosis of cancer. miRs that have been confirmed to be related to the early diagnosis and differential diagnosis of MF include miR-93, miR-146a, 146b-5p, miR-342–3p, miR-16, miR-181, miR-203, and miR-205 (24, 30, 34, 71). Specifically, miR-93 not only plays a role in the pathogenesis of MF but can also be used as a specific biomarker for the diagnosis of MF. The significant downregulation of miR-93 can be used for the early diagnosis of early challenging cases (32).

Although some single miRs do not have independent diagnostic value in MF, the specific combinations of miRs may achieve good diagnostic ability. As mentioned before, miR-155 plays an important role in the pathogenesis and progression of MF. However, given that higher miR-155 expression was also observed in T-cell-rich benign inflammatory dermatoses compared with early MF and folliculotropic MF (FMF), it cannot be used as a separate biomarker to distinguish early MF from benign inflammation dermatosis (18, 27). Interestingly, the combination of miR-155 with specific miRs has been proven to be of great value in differential diagnosis and early diagnosis in multiple studies. Ralfkiaer et al. developed a three-miR classifier composed of miR-155, miR-203, and miR-205 that can distinguish CTCL from benign inflammatory dermatosis with an accuracy of more than 90% (24). The strength of this classifier was also confirmed in subsequent independent cohort studies (71). Moreover, in view of the stability of miRs in serum or plasma, Dusilkova et al. established a plasma multiple miR classifier based on the upregulation of miR-155 and downregulation of miR-203/miR-205 to detect CTCL with 100% specificity and 94% sensitivity, making routine clinical monitoring possible in the future (19). In addition, other investigations have suggested that a four-miR classifier composed of miR-181a, miR-146a, miR-222, and miR-26a could discriminate tumoral MF from benign inflammatory disease (34). Moreover, miR-181a and miR-146a may be used as specific biomarkers of MF and are significantly upregulated in both early and advanced MF (30, 34, 36). Importantly, there may be racial differences in the application scope of this classifier. An additional 5-miR diagnostic classifier, including miR-130b, miR-142–3p, miR-155, miR-200b, miR-243, and miR-203, was established for the diagnosis and prognosis of CTCL in a study of an Asian population (16). Taken together, the diagnostic classifier combined with multiple miRs has better diagnostic intensity and accuracy and is expected to be a valuable adjunct in future clinical work.

### Is miR Differentially Expressed in Different Variants of MF?

According to its clinicopathologic characteristics, MF can be divided into several variants (72). Folliculotropic MF (FMF) is a rare variant of MF with distinct clinicopathological features. The clinical course and treatment response varies according to different stage of disease (early- and advanced-stage) (73, 74). Specifically, patients with FMF presenting with only patches and/or follicular papules (early-stage) had a favorable prognosis with a 5-year overall survival (OS) of 92%, while patients with FMF presenting with tumors and/or nodules (advanced-stage) had a 5-year OS of 50%. Additionally, a small number of MF patients may undergo large cell transformation (TMF), which is characterized by an aggressive clinical course and refraction to systemic therapies including anthracyclines, bexarotene, methotrexate with a median survival of 18.4 to 24 months (75–77). However, the molecular background of FMF and TMF has not been fully elucidated. Marosvári D et al. showed for the first time in 2015 that miR-93–5p, miR-181a, and miR-34a were significantly upregulated in FMF and TMF. Overexpression of miR-155 and miR-223 was also observed in FMF (13). Additionally, Garaicoa et al. compared the miR expression profile among tumoral MF, FMF, and TMF and found that the expression levels of miR-19b, miR-92a, and miR-155 in FMF and TMF were higher than those in tumoral MF (23). In recent years, some scholars have proposed that according to different clinicopathological features, FMF can be categorized in early stage with indolent clinical course and advanced/tumoral stage requiring aggressive treatment (74, 78). Atzmony et al. found that there was a significant difference in miR-155 expression between early and tumoral FMF, but there was no significant difference in miR-155 expression between early FMF and MF or between tumoral FMF and MF (27). To some extent, this finding confirms that there might be two different stages of FMF, but the relationship between MF and FMF in different stages still needs further research and exploration to provide a theoretical basis for personalized treatment of different stages and subtypes of MF. In addition, the study showed that miR-181b and miR-93–5p were highly expressed in the TMF, while the level of miR-155 was not significantly increased, indicating that miR-181b and miR-93–5p may play a role in the pathogenesis of the TMF, while the regulation of miR-155-related gene expression may not be involved in large cell transformation (13).

In some special cases, it is difficult to distinguish erythroderma MF (eMF) from Sézary Syndrome (SS) clinically and histologically. Due to the differences in treatment recommendations and prognosis, it is necessary to distinguish between eMF and SS. Rittig et al. found that there was a significant difference in the miR expression profile between eMF and SS. In particular, the expression levels of miR-106b, miR-155, and miR-21 in eMF were significantly lower than those in SS (14).

Unilesional MF is characterized by a solitary erythematous patch or plaque located on the trunk and upper extremities clinically and is histologically indistinguishable from typical MF. Unlike early MF, unilesional MF can maintain a benign clinical course without any treatment, and there is no obvious recurrence after treatment. Studies have shown that the miR expression profile of unilesional MF is different from that of early MF. The former has a high level of miR-17~92 members and is accompanied by Th1 skewing (38). The antitumor activity of miR-17~92 has been confirmed in numerous previous studies (79, 80). These findings suggest that there is a strong reactive T cell immune response in unilesional MF, which may explain the locality of the disease.

### Existing miR-Based Therapeutics and Potential Treatment Options in MF

Local and systemic therapies available for MF have reduced tumor burden and improved quality of life. However, classic regimens based on anthracycline or nucleoside analogs can only obtain a short-lived response and have had limited impact on the survival of patients with advanced MF (81, 82). Therefore, advances in MF treatment research are focused on identifying new pharmacological targets. Current clinical trials show that miR-based treatment seems to be feasible (83). Given the important role of miR-155 in MF, Moyal et al. found that miR-155 promotes tumor growth in xenografted MF mice by reducing apoptosis. Anti-miR-155 can be used as monotherapy or in combination with apoptosis therapy and cell cycle checkpoint inhibitors to improve the effectiveness of MF therapy (20). Indeed, clinical trials were conducted to test the therapeutic efficacy of a miR-155-targeting nucleic acid modification inhibitor called cobomarsen (MRG-106) in MF in 2018 (26). Cobomarsen can reduce the expression of multiple gene pathways related to cell survival by blocking miR-155 and finally reduce cell proliferation and activate apoptosis. Therefore, it can be suggested that cobomarsen can potentially be used as a therapeutic agent for MF (26). Surprisingly, active antimicrobial therapy can inhibit the activity of MF by affecting the expression of miR-155 (22, 25). Indeed, Duvich et al. reported that the combination of antibiotics, a germicidal whirlpool bath system, and steroids has a significant effect on patients with SS. *In vitro* experiments demonstrated that active antibiotic therapy may inhibit malignant T cell proliferation in advanced CTCL by inhibiting the staphylococcal enterotoxin (SE)-mediated bystander T cell response (35). Recent studies have further shown that this inhibitory effect may be achieved by partially reversing SE-induced pathological processes involving STAT5 and miR-155 (21). Notably, *Staphylococcus aureus* is also present to a greater extent in skin lesions in patients with MF than in patients with non-lesional or healthy skin. miR-155 may be associated with secondary skin infection in patients with MF. Additionally, the involvement of other infectious agents in MF, such as *Borrelia burgdorferi, Chlamydophila*, have been described (84). A previous study by Tothova et al. found that *Borrelia* might exert its causative role in MF through a chronic type-1 immune response with interferon-γ production (85). Besides, evidence of type C pneumonia in MF biopsies suggests an association between *Chlamydophila* infection and MF development (86). However, further studies are needed to assess the efficacy of anti-*Borrelia* therapy in treating *Borrelia*-positive MF and verify the correlation between *Chlamydophila* and MF in larger cohorts. In view of the important role of antimicrobial therapy and miRs in MF, the combination of miR-based therapy and antimicrobial therapy may produce synergistic therapeutic effects and improve the therapeutic efficacy of patients with MF, especially for advanced MF with large-area involvement.

Interestingly, miR-106b was previously known to provide the strongest prognostic prediction of a high risk of progression (87). Given that miR-106b is also highly expressed in the early stage of MF, the development of miR-106b inhibitors applied in the early stage of the disease may prevent or delay disease progression (36). In addition, inhibition of miR-93 resulted in decreased proliferation of malignant T cell lines. The effect of this reduction in proliferation was similar to that observed following inhibition of miR-155 (11, 29, 88). Thus, anti-miR-93 could be a valuable therapeutic agent for patients with MF. Furthermore, researchers found that miR-122 was overexpressed in advanced MF, reducing sensitivity to chemotherapy-induced apoptosis through signal transduction circuits involved in Akt activation and p53 inhibition. This finding provides a new idea for the use of chemotherapy sensitizers in advanced MF (41). In addition to the development of the abovementioned miR-based therapeutic drugs, the miR expression spectrum can also predict treatment response. Studies have shown that MF patients with rapidly elevated levels of miR-223, miR-191, and miR-342 after extracorporeal blood collection are more likely to show good clinical responses after 6–12 months of treatment (42).

### miR May Play an Important Role in Predicting Prognosis in MF

miRs not only contribute to increasing the accuracy of diagnosis but can also be used to predict prognosis. Several studies have emphasized that miRs can be used as a potential prognostic biomarker in MF. For instance, the miRs of the let-7 family are downregulated in advanced and metastatic MF. The prognosis of patients with low expression of let-7a was worse than that of other patients (15). Of note, although multiple studies have shown that miR-155 can be used as a prognostic indicator of CTCL, its prognostic value in MF and the relationship between the intensity of miR-155 expression and disease stage are discrepant (16, 17, 26). Shen et al. found that miR-155 was associated with worse clinical outcomes in an Asian population with a total of 158 CTCLs (including MF, cutaneous anaplastic large cell lymphoma, peripheral T cell lymphoma and NK/T cell lymphoma). However, excluding other types of CTCL than MF, the expression of miR-155 had no significant correlation with the overall survival of patients with different stages of MF (16). The inconsistency in the above findings may be attributed to the different ethnic backgrounds of the patients and varied numbers of patients with different disease stages enrolled in the above studies. Comparatively, miR-200b is significantly associated with the overall survival of MF patients regardless of disease stage (16). High miR-200b expression implied favorable prognosis. Taken together, there may be differences in prognostic markers among subgroups of CTCLs, and it is necessary to develop specific prognostic biomarkers for MF patients.

Early identification of patients at a higher risk of progression may facilitate more individualized treatment of these patients. Therefore, it is worth drawing attention to the prognostic stratification of patients with early MF. Lindahl et al. developed and verified a 3-miR prognostic classifier based on miR-106b-5p, miR-148a-3p, and miR-338–3p, which can successfully divide patients into high- and low-risk groups for disease progression. This 3-miR classifier may be an effective tool to predict the progression of early MF at the time of diagnosis and has important prognostic value (36). Among them, miR-106b is the most powerful prognostic marker of disease progression in MF. As the disease progresses, miR-106b can regulate the expression of the tumor suppressor genes p21 and thioredoxin-interacting protein (TXNIP) and promote the proliferation of tumor cells in MF (37). Additionally, there were also differences in the expression levels of miR-16, miR-93, and miR-106a between progressive and nonprogressive patients (30). Of note, there may also be racial differences in the application scope of this classifier. Shen et al. verified that miR-155 and miR-200b can be used as effective predictors of clinical outcome in Asian populations (16). Therefore, it is necessary to develop and validate prognostic miR classifiers among populations with different racial backgrounds.

### Future Perspectives of Exosomal miR-Based Therapeutics in MF

In addition to the tumor cells themselves, the malignant tumor phenotype can also transmit genetic information, including miRs, to other cells in the tumor microenvironment through exosomes, which can promote proliferation, angiogenesis, metastasis, and drug resistance. Moyal et al. first identified miR-155 and miR-1246 in exosomes derived from MF cell lines in 2021. It was confirmed that exomiR-155 can promote the motility of MF cell lines *in vitro*. In addition, exomiR-1246 expression levels appeared to be correlated with MF staging, and were significantly higher in plaque/tumor MF patients than in healthy population (28). Moreover, exosomal miRs can affect extracellular matrix (ECM) and immune system activation and recruitment to promote tumor cell survival. Furthermore, exosomal miRs can be used as promising noninvasive biomarkers and potential targeting factors and delivery vehicles for tumor diagnosis and treatment. Therefore, exosomal miR-based therapeutics are a potential feasible candidate therapy for MF.

## Conclusion

MF is the most common cutaneous T-cell lymphoma. The main problem in the diagnosis and treatment of MF is the inefficient methods of early diagnosis and short-lived response to classical chemotherapy. The emergence of the role of miR in cancer progression has prompted us to elucidate the prospects of miR as new therapeutic target. Several studies have identified them as potential diagnostic and prognostic biomarkers in MF. The identification of these new gene regulatory targets opens up a new field for the diagnosis, treatment and prognosis of MF. However, aberrant miR expression have been detected in a variety inflammatory disease and malignancies, and disease-specific miR remains as one of the most challenging issues. Additionally, the functions of partial miRs in MF remain controversial and further investigation and validation in larger cohorts are needed. Also, miR-based therapeutics in CTCL is still in budding stages, the reliable and targeted site-specific delivery of miR might be a major obstacle to the use of miR-based therapy. Thus, it is urgently needed to overcome the above obstacles before development of novel miR-based therapeutics strategy.

## Author Contributions

PFW designed and wrote this article. YX made a contribution to revision. LW designed the whole project and revised the final manuscript. All authors contributed to the article and approved the submitted version.

## Conflict of Interest

The authors declare that the research was conducted in the absence of any commercial or financial relationships that could be construed as a potential conflict of interest.

## Publisher’s Note

All claims expressed in this article are solely those of the authors and do not necessarily represent those of their affiliated organizations, or those of the publisher, the editors and the reviewers. Any product that may be evaluated in this article, or claim that may be made by its manufacturer, is not guaranteed or endorsed by the publisher.

## References

[1] Saunes M Nilsen TI Johannesen TB. Incidence of Primary Cutaneous T-Cell Lymphoma in Norway. Br J Dermatol (2009) 160(2):376–9. doi: 10.1111/j.1365-2133.2008.08852.x 18808419

[2] Hristov AC Tejasvi T Wilcox RA. Mycosis Fungoides and Sezary Syndrome: 2019 Update on Diagnosis, Risk-Stratification, and Management. Am J Hematol (2019) 94(9):1027–41. doi: 10.1002/ajh.25577 31313347

[3] Agar NS Wedgeworth E Crichton S Mitchell TJ Cox M Ferreira S. Survival Outcomes and Prognostic Factors in Mycosis Fungoides/Sezary Syndrome: Validation of the Revised International Society for Cutaneous Lymphomas/European Organisation for Research and Treatment of Cancer Staging Proposal. J Clin Oncol (2010) 28(31):4730–9. doi: 10.1200/JCO.2009.27.7665 20855822

[4] Wong HK Mishra A Hake T Porcu P. Evolving Insights in the Pathogenesis and Therapy of Cutaneous T-Cell Lymphoma (Mycosis Fungoides and Sezary Syndrome). Br J Haematol (2011) 155(2):150–66. doi: 10.1111/j.1365-2141.2011.08852.x PMC4309373 21883142

[5] Thurber SE Zhang B Kim YH Schrijver I Zehnder J Kohler S. T-Cell Clonality Analysis in Biopsy Specimens From Two Different Skin Sites Shows High Specificity in the Diagnosis of Patients With Suggested Mycosis Fungoides. J Am Acad Dermatol (2007) 57(5):782–90. 10.1016/j.jaad.2007.06.004 17646032

[6] Hodges E Krishna MT Pickard C Smith JL. Diagnostic Role of Tests for T Cell Receptor (TCR) Genes. J Clin Pathol (2003) 56(1):1–11. doi: 10.1136/jcp.56.1.1 12499424 PMC1769865

[7] Ponti R Fierro MT Quaglino P Lisa B Paola F Michela O. TCRgamma-Chain Gene Rearrangement by PCR-Based GeneScan: Diagnostic Accuracy Improvement and Clonal Heterogeneity Analysis in Multiple Cutaneous T-Cell Lymphoma Samples. J Invest Dermatol (2008) 128(4):1030–8. doi: 10.1038/sj.jid.5701109 17989737

[8] Phyo ZH Shanbhag S Rozati S. Update on Biology of Cutaneous T-Cell Lymphoma. Front Oncol (2020) 10:765. doi: 10.3389/fonc.2020.00765 32477957 PMC7235328

[9] Rupaimoole R Slack FJ. MicroRNA Therapeutics: Towards a New Era for the Management of Cancer and Other Diseases. Nat Rev Drug Discovery (2017) 16(3):203–22. doi: 10.1038/nrd.2016.246 28209991

[10] Calin GA Croce CM. MicroRNA Signatures in Human Cancers. Nat Rev Cancer (2006) 6(11):857–66. doi: 10.1038/nrc1997 17060945

[11] Kopp KL Ralfkiaer U Gjerdrum LM Helvad R Pedersen IH Litman T. STAT5-Mediated Expression of Oncogenic miR-155 in Cutaneous T-Cell Lymphoma. Cell Cycle (2013) 12(12):1939–47. doi: 10.4161/cc.24987 PMC3735708 23676217

[12] Li N Miao Y Shan Y Liu B Li Y Zhao L. MiR-106b and miR-93 Regulate Cell Progression by Suppression of PTEN *via* PI3K/Akt Pathway in Breast Cancer. Cell Death Dis (2017) 8(5):e2796. doi: 10.1038/cddis.2017.119 28518139 PMC5520687

[13] Marosvari D Teglasi V Csala I Marschalko M Bodor C Timar B. Altered microRNA Expression in Folliculotropic and Transformed Mycosis Fungoides. Pathol Oncol Res (2015) 21(3):821–5. doi: 10.1007/s12253-015-9897-8 25698383

[14] Rittig AH Lindahl LM Johansen C Celis P Odum N Iversen L. The MicroRNA Expression Profile Differs Between Erythrodermic Mycosis Fungoides and Sezary Syndrome. Acta Derm Venereol (2019) 99(12):1148–53. doi: 10.2340/00015555-3306 31453630

[15] van Kester MS Ballabio E Benner MF Chen XH Saunders NJ van der Fits L. miRNA Expression Profiling of Mycosis Fungoides. Mol Oncol (2011) 5(3):273–80. doi: 10.1016/j.molonc.2011.02.003 PMC5528293 21406335

[16] Shen X Wang B Li K Wang L Zhao X Xue F. MicroRNA Signatures in Diagnosis and Prognosis of Cutaneous T-Cell Lymphoma. J Invest Dermatol (2018) 138(9):2024–32. doi: 10.1016/j.jid.2018.03.1500 29559342

[17] Maj J Jankowska-Konsur A Sadakierska-Chudy A Noga L Reich A. Altered microRNA Expression in Mycosis Fungoides. Br J Dermatol (2012) 166(2):331–6. doi: 10.1111/j.1365-2133.2011.10669.x 21966986

[18] Moyal L Barzilai A Gorovitz B Hirshberg A Amariglio N Jacob-Hirsch J. miR-155 Is Involved in Tumor Progression of Mycosis Fungoides. Exp Dermatol (2013) 22(6):431–3. doi: 10.1111/exd.12161 23711069

[19] Dusilkova N Basova P Polivka J Kodet O Kulvait V Pesta M. Plasma miR-155, miR-203, and miR-205 Are Biomarkers for Monitoring of Primary Cutaneous T-Cell Lymphomas. Int J Mol Sci (2017) 18(10). doi: 10.3390/ijms18102136 PMC5666818 29036928

[20] Moyal L Yehezkel S Gorovitz B Keren A Gilhar A Lubin I. Oncogenic Role of microRNA-155 in Mycosis Fungoides: An *In Vitro* and Xenograft Mouse Model Study. Br J Dermatol (2017) 177(3):791–800. doi: 10.1111/bjd.15422 28256712

[21] Willerslev-Olsen A Gjerdrum LMR Lindahl LM Buus TB Pallesen EMH Gluud M. Staphylococcus Aureus Induces Signal Transducer and Activator of Transcription 5dependent miR-155 Expression in Cutaneous T-Cell Lymphoma. J Invest Dermatol (2021). doi: 10.1016/j.jid.2021.01.038 33862068

[22] Lewis DJ Holder BB Duvic M. The "Duvic Regimen" for Erythrodermic Flares Secondary to Staphylococcus Aureus in Mycosis Fungoides and Sezary Syndrome. Int J Dermatol (2018) 57(1):123–4. doi: 10.1111/ijd.13832 29152728

[23] Garaicoa FH Roisman A Arias M Trila C Fridmanis M Abeldano A. Genomic Imbalances and microRNA Transcriptional Profiles in Patients With Mycosis Fungoides. Tumour Biol (2016) 37(10):13637–47. doi: 10.1007/s13277-016-5259-8 27473081

[24] Ralfkiaer U Hagedorn PH Bangsgaard N Lovendorf MB Ahler CB Svensson L. Diagnostic microRNA Profiling in Cutaneous T-Cell Lymphoma (CTCL). Blood (2011) 118(22):5891–900. doi: 10.1182/blood-2011-06-358382 PMC3342856 21865341

[25] Talpur R Bassett R Duvic M. Prevalence and Treatment of Staphylococcus Aureus Colonization in Patients With Mycosis Fungoides and Sezary Syndrome. Br J Dermatol (2008) 159(1):105–12. doi: 10.1111/j.1365-2133.2008.08612.x 18489588

[26] Seto AG Beatty X Lynch JM Hermreck M Tetzlaff M Duvic M. Cobomarsen, an Oligonucleotide Inhibitor of miR-155, Co-Ordinately Regulates Multiple Survival Pathways to Reduce Cellular Proliferation and Survival in Cutaneous T-Cell Lymphoma. Br J Haematol (2018) 183(3):428–44. doi: 10.1111/bjh.15547 30125933

[27] Atzmony L Moyal L Feinmesser M Gorovitz B Hirshberg A Amitay-Laish I. Stage-Dependent Increase in Expression of miR-155 and Ki-67 and Number of Tumour-Associated Inflammatory Cells in Folliculotropic Mycosis Fungoides. Acta Derm Venereol (2020) 100(15):adv00230. doi: 10.2340/00015555-3578 32556361 PMC9207631

[28] Moyal L Arkin C Gorovitz-Haris B Querfeld C Rosen S Knaneh J. Mycosis Fungoides-Derived Exosomes Promote Cell Motility and Are Enriched With microRNA-155 and microRNA-1246, and Their Plasma-Cell-Free Expression May Serve as a Potential Biomarker for Disease Burden. Br J Dermatol (2021). doi: 10.1111/bjd.20519 PMC8864770 34053079

[29] Gluud M Fredholm S Blumel E Willerslev-Olsen A Buus TB Nastasi C. MicroRNA-93 Targets P21 and Promotes Proliferation in Mycosis Fungoides T Cells. Dermatology (2021) 237(2):277–82. doi: 10.1159/000505743 32335549

[30] Ralfkiaer U Lindahl LM Litman T Gjerdrum LM Ahler CB Gniadecki R. MicroRNA Expression in Early Mycosis Fungoides Is Distinctly Different From Atopic Dermatitis and Advanced Cutaneous T-Cell Lymphoma. Anticancer Res (2014) 34(12):7207–17. 25503151

[31] Hanahan D Weinberg RA. The Hallmarks of Cancer. Cell (2000) 100(1):57–70. doi: 10.1016/s0092-8674(00)81683-9 10647931

[32] Talaat IM Abdelmaksoud RE Guimei M Agamia NF Nugud A El-Serafi AT. Potential Role for microRNA-16 (miR-16) and microRNA-93 (miR-93) in Diagnosis and Prediction of Disease Progression in Mycosis Fungoides in Egyptian Patients. PloS One (2019) 14(10):e0224305. doi: 10.1371/journal.pone.0224305 31648231 PMC6812867

[33] McGirt LY Adams CM Baerenwald DA Zwerner JP Zic JA Eischen CM. miR-223 Regulates Cell Growth and Targets Proto-Oncogenes in Mycosis Fungoides/Cutaneous T-Cell Lymphoma. J Invest Dermatol (2014) 134(4):1101–7. doi: 10.1038/jid.2013.461 PMC3961555 24304814

[34] Manso R Martinez-Magunacelaya N Erana-Tomas I Monsalvez V Rodriguez-Peralto JL Ortiz-Romero PL. Mycosis Fungoides Progression Could be Regulated by microRNAs. PloS One (2018) 13(6):e0198477. doi: 10.1371/journal.pone.0198477 29894486 PMC5997347

[35] Lindahl LM Willerslev-Olsen A Gjerdrum LMR Nielsen PR Blumel E Rittig AH. Antibiotics Inhibit Tumor and Disease Activity in Cutaneous T-Cell Lymphoma. Blood (2019) 134(13):1072–83. doi: 10.1182/blood.2018888107 PMC6764271 31331920

[36] Lindahl LM Besenbacher S Rittig AH Celis P Willerslev-Olsen A Gjerdrum LMR. Prognostic miRNA Classifier in Early-Stage Mycosis Fungoides: Development and Validation in a Danish Nationwide Study. Blood (2018) 131(7):759–70. doi: 10.1182/blood-2017-06-788950 29208599

[37] Lindahl LM Gluud M Emmanuel T Thomsen EA Hu T Rittig AH. MicroRNA-106b Regulates Expression of the Tumour Suppressors P21 and TXNIP and Promotes Tumour Cell Proliferation in Mycosis Fungoides. Acta Derm Venereol (2020) 100(16):adv00270. doi: 10.2340/00015555-3574 32556351 PMC9234987

[38] Moyal L Gorovitz-Haris B Yehezkel S Jacob-Hirsch J Bershtein V Barzilai A. Unilesional Mycosis Fungoides Is Associated With Increased Expression of microRNA-17~92 and T Helper 1 Skewing. Br J Dermatol (2019) 180(5):1123–34. doi: 10.1111/bjd.17425 30431147

[39] Sibbesen NA Kopp KL Litvinov IV Jonson L Willerslev-Olsen A Fredholm S. Jak3, STAT3, and STAT5 Inhibit Expression of miR-22, a Novel Tumor Suppressor microRNA, in Cutaneous T-Cell Lymphoma. Oncotarget (2015) 6(24):20555–69. doi: 10.18632/oncotarget.4111 PMC4653025 26244872

[40] Rittig AH Johansen C Celis P Odum N Litman T Woetmann A. Suppressed microRNA-195-5p Expression in Mycosis Fungoides Promotes Tumor Cell Proliferation. Exp Dermatol (2020). doi: 10.1111/exd.14124 32492224

[41] Manfe V Biskup E Rosbjerg A Kamstrup M Skov AG Lerche CM. miR-122 Regulates P53/Akt Signalling and the Chemotherapy-Induced Apoptosis in Cutaneous T-Cell Lymphoma. PloS One (2012) 7(1):e29541. doi: 10.1371/journal.pone.0029541 22235305 PMC3250447

[42] McGirt LY Baerenwald DA Vonderheid EC Eischen CM. Early Changes in miRNA Expression Are Predictive of Response to Extracorporeal Photopheresis in Cutaneous T-Cell Lymphoma. J Eur Acad Dermatol Venereol (2015) 29(11):2269–71. doi: 10.1111/jdv.12571 PMC4831701 24909834

[43] Morris KV Chan SW Jacobsen SE Looney DJ. Small Interfering RNA-Induced Transcriptional Gene Silencing in Human Cells. Science (2004) 305(5688):1289–92. doi: 10.1126/science.1101372 15297624

[44] Duffy J Padovani F Brunetti G Noy P Certa U Hegner M. Towards Personalised Rapid Label Free miRNA Detection for Cancer and Liver Injury Diagnostics in Cell Lysates and Blood Based Samples. Nanoscale (2018) 10(26):12797–804. doi: 10.1039/c8nr03604g 29947396

[45] Jørgensen S Paulsen IW Hansen JW Tholstrup D Hother C Sørensen E. The Value of Circulating microRNAs for Early Diagnosis of B-Cell Lymphoma: A Case-Control Study on Historical Samples. Sci Rep (2020) 10(1):9637. doi: 10.1038/s41598-020-66062-1 32541886 PMC7295742

[46] Wang Y Yin W Lin Y Yin K Zhou L Du Y. Downregulated Circulating microRNAs After Surgery: Potential Noninvasive Biomarkers for Diagnosis and Prognosis of Early Breast Cancer. Cell Death Discovery (2018) 4:21. doi: 10.1038/s41420-018-0089-7 PMC6078958 30109140

[47] Li Z Rana TM. Therapeutic Targeting of microRNAs: Current Status and Future Challenges. Nat Rev Drug Discovery (2014) 13(8):622–38. doi: 10.1038/nrd4359 25011539

[48] Papadavid E Braoudaki M Bourdakou M Lykoudi A Nikolaou V Tounta G. Aberrant microRNA Expression in Tumor Mycosis Fungoides. Tumour Biol (2016) 37(11):14667–75. doi: 10.1007/s13277-016-5325-2 27623940

[49] Gluud M Willerslev-Olsen A Gjerdrum LMR Lindahl LM Buus TB Andersen MH. MicroRNAs in the Pathogenesis, Diagnosis, Prognosis and Targeted Treatment of Cutaneous T-Cell Lymphomas. Cancers (Basel) (2020) 12(5). doi: 10.3390/cancers12051229 PMC7281391 32414221

[50] Sandoval J Diaz-Lagares A Salgado R Servitje O Climent F Ortiz-Romero PL. MicroRNA Expression Profiling and DNA Methylation Signature for Deregulated microRNA in Cutaneous T-Cell Lymphoma. J Invest Dermatol (2015) 135(4):1128–37. doi: 10.1038/jid.2014.487 25405321

[51] Merkel O Hamacher F Griessl R Grabner L Schiefer AI Prutsch N. Oncogenic Role of miR-155 in Anaplastic Large Cell Lymphoma Lacking the T(2;5) Translocation. J Pathol (2015) 236(4):445–56. doi: 10.1002/path.4539 PMC4557053 25820993

[52] Narducci MG Arcelli D Picchio MC Lazzeri C Pagani E Sampogna F. MicroRNA Profiling Reveals That miR-21, Mir486 and miR-214 Are Upregulated and Involved in Cell Survival in Sezary Syndrome. Cell Death Dis (2011) 2:e151. doi: 10.1038/cddis.2011.32 21525938 PMC3122063

[53] Ballabio E Mitchell T van Kester MS Taylor S Dunlop HM Chi J. MicroRNA Expression in Sezary Syndrome: Identification, Function, and Diagnostic Potential. Blood (2010) 116(7):1105–13. doi: 10.1182/blood-2009-12-256719 PMC2938132 20448109

[54] Valencak J Schmid K Trautinger F Wallnöfer W Muellauer L Soleiman A. High Expression of Dicer Reveals a Negative Prognostic Influence in Certain Subtypes of Primary Cutaneous T Cell Lymphomas. J Dermatol science (2011) 64(3):185–90. doi: 10.1016/j.jdermsci.2011.08.011 21937200

[55] Ahmadvand M Eskandari M Pashaiefar H Yaghmaie M Manoochehrabadi S Khakpour G. Over Expression of Circulating miR-155 Predicts Prognosis in Diffuse Large B-Cell Lymphoma. Leuk Res (2018) 70:45–8. doi: 10.1016/j.leukres.2018.05.006 29807272

[56] Patel GK Khan MA Bhardwaj A Srivastava SK Zubair H Patton MC. Exosomes Confer Chemoresistance to Pancreatic Cancer Cells by Promoting ROS Detoxification and miR-155-Mediated Suppression of Key Gemcitabine-Metabolising Enzyme, DCK. Br J Cancer (2017) 116(5):609–19. doi: 10.1038/bjc.2017.18 PMC5344296 28152544

[57] Volinia S Calin GA Liu CG Ambs S Cimmino A Petrocca F. A microRNA Expression Signature of Human Solid Tumors Defines Cancer Gene Targets. Proc Natl Acad Sci USA (2006) 103(7):2257–61. doi: 10.1073/pnas.0510565103 PMC1413718 16461460

[58] Mirvish ED Pomerantz RG Geskin LJ. Infectious Agents in Cutaneous T-Cell Lymphoma. J Am Acad Dermatol (2011) 64(2):423–31. doi: 10.1016/j.jaad.2009.11.692 PMC3954537 20692726

[59] Mirvish JJ Pomerantz RG Falo LD Jr. Geskin LJ. Role of Infectious Agents in Cutaneous T-Cell Lymphoma: Facts and Controversies. Clin Dermatol (2013) 31(4):423–31. doi: 10.1016/j.clindermatol.2013.01.009 23806159

[60] Katona TM Smoller BR Webb AL Hattab EM Khalil A Hiatt KM. Expression of PTEN in Mycosis Fungoides and Correlation With Loss of Heterozygosity. Am J Dermatopathol (2013) 35(5):555–60. doi: 10.1097/DAD.0b013e318276cc68 23715078

[61] Hsu SD Tseng YT Shrestha S Lin YL Khaleel A Chou CH. Mirtarbase Update 2014: An Information Resource for Experimentally Validated miRNA-Target Interactions. Nucleic Acids Res (2014) 42(Database issue):D78–85. doi: 10.1093/nar/gkt1266 PMC3965058 24304892

[62] Williams A Henao-Mejia J Harman CC Flavell RA. miR-181 and Metabolic Regulation in the Immune System. Cold Spring Harb Symp Quant Biol (2013) 78:223–30. doi: 10.1101/sqb.2013.78.020024 24163395

[63] Nicoloso MS Kipps TJ Croce CM Calin GA. MicroRNAs in the Pathogeny of Chronic Lymphocytic Leukaemia. Br J Haematol (2007) 139(5):709–16. doi: 10.1111/j.1365-2141.2007.06868.x 18021085

[64] Bottoni A Piccin D Tagliati F Luchin A Zatelli MC degli Uberti EC. miR-15a and miR-16-1 Down-Regulation in Pituitary Adenomas. J Cell Physiol (2005) 204(1):280–5. doi: 10.1002/jcp.20282 15648093

[65] Aronovich A Moyal L Gorovitz B Amitay-Laish I Naveh HP Forer Y. Cancer-Associated Fibroblasts in Mycosis Fungoides Promote Tumor Cell Migration and Drug Resistance Through CXCL12/CXCR4. J Invest Dermatol (2021) 141(3):619–627 e2. doi: 10.1016/j.jid.2020.06.034 32795528

[66] Odum N. Deregulated Signalling and Inflammation in Cutaneous T-Cell Lymphoma. Br J Dermatol (2020) 182(1):16–7. doi: 10.1111/bjd.18353 31390055

[67] Li B Song Y Liu TJ Cui YB Jiang Y Xie ZS. miRNA-22 Suppresses Colon Cancer Cell Migration and Invasion by Inhibiting the Expression of T-Cell Lymphoma Invasion and Metastasis 1 and Matrix Metalloproteinases 2 and 9. Oncol Rep (2013) 29(5):1932–8. doi: 10.3892/or.2013.2300 23440286

[68] Pimpinelli N Olsen EA Santucci M Vonderheid E Haeffner AC Stevens S. Defining Early Mycosis Fungoides. J Am Acad Dermatol (2005) 53(6):1053–63. doi: 10.1016/j.jaad.2005.08.057 16310068

[69] Keehn CA Belongie IP Shistik G Fenske NA Glass LF. The Diagnosis, Staging, and Treatment Options for Mycosis Fungoides. Cancer Control (2007) 14(2):102–11. doi: 10.1177/107327480701400203 17387295

[70] Wilcox RA. Cutaneous T-Cell Lymphoma: 2016 Update on Diagnosis, Risk-Stratification, and Management. Am J Hematol (2016) 91(1):151–65. doi: 10.1002/ajh.24233 PMC4715621 26607183

[71] Marstrand T Ahler CB Ralfkiaer U Clemmensen A Kopp KL Sibbesen NA. Validation of a Diagnostic microRNA Classifier in Cutaneous T-Cell Lymphomas. Leuk Lymphoma (2014) 55(4):957–8. doi: 10.3109/10428194.2013.815352 23772646

[72] Swerdlow SH Campo E Pileri SA Harris NL Stein H Siebert R. The 2016 Revision of the World Health Organization Classification of Lymphoid Neoplasms. Blood (2016) 127(20):2375–90. doi: 10.1182/blood-2016-01-643569 PMC4874220 26980727

[73] van Santen S van Doorn R Neelis KJ Daniëls LA Horváth B Bruijn MS. Recommendations for Treatment in Folliculotropic Mycosis Fungoides: Report of the Dutch Cutaneous Lymphoma Group. Br J Dermatol (2017) 177(1):223–8. doi: 10.1111/bjd.15355 28132406

[74] van Santen S Roach RE van Doorn R Horvath B Bruijn MS Sanders CJ. Clinical Staging and Prognostic Factors in Folliculotropic Mycosis Fungoides. JAMA Dermatol (2016) 152(9):992–1000. doi: 10.1001/jamadermatol.2016.1597 27276223

[75] Vergier B de Muret A Beylot-Barry M Vaillant L Ekouevi D Chene G. Transformation of Mycosis Fungoides: Clinicopathological and Prognostic Features of 45 Cases. French Study Group of Cutaneious Lymphomas. Blood (2000) 95(7):2212–8. 10733487

[76] Benner MF Jansen PM Vermeer MH Willemze R. Prognostic Factors in Transformed Mycosis Fungoides: A Retrospective Analysis of 100 Cases. Blood (2012) 119(7):1643–9. doi: 10.1182/blood-2011-08-376319 22160616

[77] Lansigan F Horwitz SM Pinter-Brown LC Carson KR Shustov AR Rosen ST. Outcomes of Patients With Transformed Mycosis Fungoides: Analysis From a Prospective Multicenter US Cohort Study. Clin Lymphoma Myeloma Leuk (2020) 20(11):744–8. doi: 10.1016/j.clml.2020.05.001 PMC8447249 32532611

[78] Hodak E Amitay-Laish I Atzmony L Prag-Naveh H Yanichkin N Barzilai A. New Insights Into Folliculotropic Mycosis Fungoides (FMF): A Single-Center Experience. J Am Acad Dermatol (2016) 75(2):347–55. doi: 10.1016/j.jaad.2016.03.009 27245278

[79] Kosaka A Ohkuri T Ikeura M Kohanbash G Okada H. Transgene-Derived Overexpression of miR-17-92 in CD8+ T-Cells Confers Enhanced Cytotoxic Activity. Biochem Biophys Res Commun (2015) 458(3):549–54. doi: 10.1016/j.bbrc.2015.02.003 PMC4355048 25677619

[80] Ohno M Ohkuri T Kosaka A Tanahashi K June CH Natsume A. Expression of miR-17-92 Enhances Anti-Tumor Activity of T-Cells Transduced With the Anti-EGFRvIII Chimeric Antigen Receptor in Mice Bearing Human GBM Xenografts. J Immunother Cancer (2013) 1:21. doi: 10.1186/2051-1426-1-21 24829757 PMC4019893

[81] Cyrenne BM Lewis JM Weed JG Carlson KR Mirza FN Foss FM. Synergy of BCL2 and Histone Deacetylase Inhibition Against Leukemic Cells From Cutaneous T-Cell Lymphoma Patients. Blood (2017) 130(19):2073–83. doi: 10.1182/blood-2017-06-792150 PMC5680613 28972015

[82] Wollina U Dummer R Brockmeyer NH Konrad H Busch JO Kaatz M. Multicenter Study of Pegylated Liposomal Doxorubicin in Patients With Cutaneous T-Cell Lymphoma. Cancer (2003) 98(5):993–1001. doi: 10.1002/cncr.11593 12942567

[83] Takahashi RU Prieto-Vila M Kohama I Ochiya T. Development of miRNA-Based Therapeutic Approaches for Cancer Patients. Cancer Sci (2019) 110(4):1140–7. doi: 10.1111/cas.13965 PMC6447849 30729639

[84] Travaglino A Varricchio S Pace M Russo D Picardi M Baldo A. Borrelia Burgdorferi in Primary Cutaneous Lymphomas: A Systematic Review and Meta-Analysis. J Dtsch Dermatol Ges (2020) 18(12):1379–84. doi: 10.1111/ddg.14289 33029842

[85] Tothova SM Bonin S Trevisan G Stanta G. Mycosis Fungoides: Is It a Borrelia Burgdorferi-Associated Disease? Br J Cancer (2006) 94(6):879–83. doi: 10.1038/sj.bjc.6602997 PMC2361364 16495924

[86] Abrams JT Balin BJ Vonderheid EC. Association Between Sézary T Cell-Activating Factor, Chlamydia Pneumoniae, and Cutaneous T Cell Lymphoma. Ann N Y Acad Sci (2001) 941:69–85. doi: 10.1111/j.1749-6632.2001.tb03712.x 11594584

[87] Liu K Pan X Peng X Zhang C Li H Guan X. Associations of High Expression of miR-106b-5p Detected From FFPE Sample With Poor Prognosis of RCC Patients. Pathol Res Pract (2019) 215(6):152391. doi: 10.1016/j.prp.2019.03.019 31076282

[88] Querfeld C Foss FM Pinter-Brown LC Porcu P William BM Pacheco T. Phase 1 Study of the Safety and Efficacy of MRG-106, a Synthetic Inhibitor of microRNA-155, in CTCL Patients. Blood (2017) 1(1):820. doi: 10.1182/blood.V130.Suppl_1.820.820

